# Home-based cardiac rehabilitation improves quality of life, aerobic capacity, and readmission rates in patients with chronic heart failure

**DOI:** 10.1097/MD.0000000000009629

**Published:** 2018-01-26

**Authors:** Yan-Wen Chen, Chi-Yen Wang, Yuan-Hui Lai, Ying-Chieh Liao, Yan-Kai Wen, Shin-Tsu Chang, Jin-Long Huang, Tsu-Juey Wu

**Affiliations:** a Department of Physical Medicine and Rehabilitation; b Cardiovascular Center, Taichung Veterans General Hospital; c Department of Health Business Administration, Hung Kuang University, Taichung; d Institute of Clinical Medicine, and Cardiovascular Research Institute, Department of Medicine, School of Medicine, National Yang-Ming University, Taipei; e Department of Physical Medicine and Rehabilitation, School of Medicine, National Defense Medical Center, Taipei, Taiwan.

**Keywords:** heart failure, home-based cardiac rehabilitation, quality of medical care

## Abstract

**Background:**

Exercise tolerance and cardiac output have a major impact on the quality of life (QOL) of patients experiencing heart failure (HF). Home-based cardiac rehabilitation can significantly improve not only exercise tolerance but also *peak oxygen uptake* (
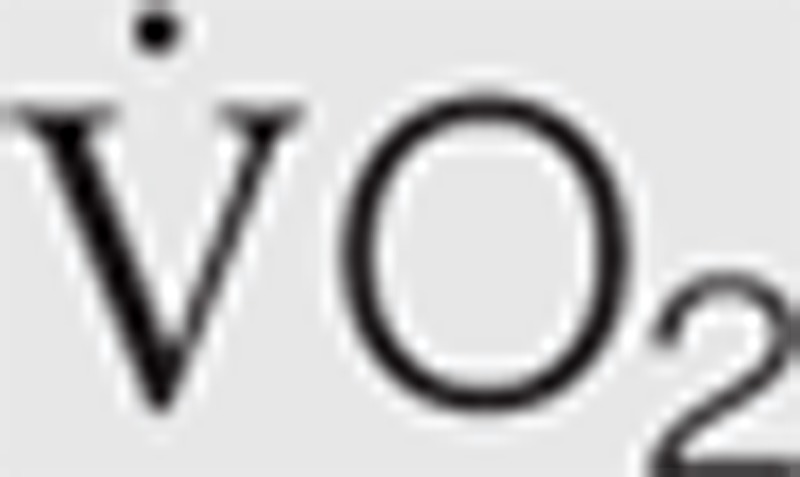
 peak), and the QOL in patients with HF. The aim of this prospective study was to evaluate the beneficial effects of home-based cardiac rehabilitation on the quality of medical care in patients with chronic HF.

**Methods:**

This study was a randomized prospective trial. HF patients with a left ventricular ejection fraction (LVEF) of less than 50% were included in this study. We randomly assigned patients to the control group (n = 18) and the interventional group (n = 19). Within the interventional group, we arranged individualized rehabilitation programs, including home-based cardiac rehabilitation, diet education, and management of daily activity over a 3-month period. Information such as general data, laboratory data, Cardiopulmonary Exercise Test (CPET) results, Six-minute Walk Test (6MWT) results, and the scores for the *Minnesota Living with Heart Failure Questionnaire (MLHFQ*) before and after the intervention, was collected from all patients in this study.

**Results:**

Patients enrolled in the home-based cardiac rehabilitation programs displayed statistically significant improvement in 
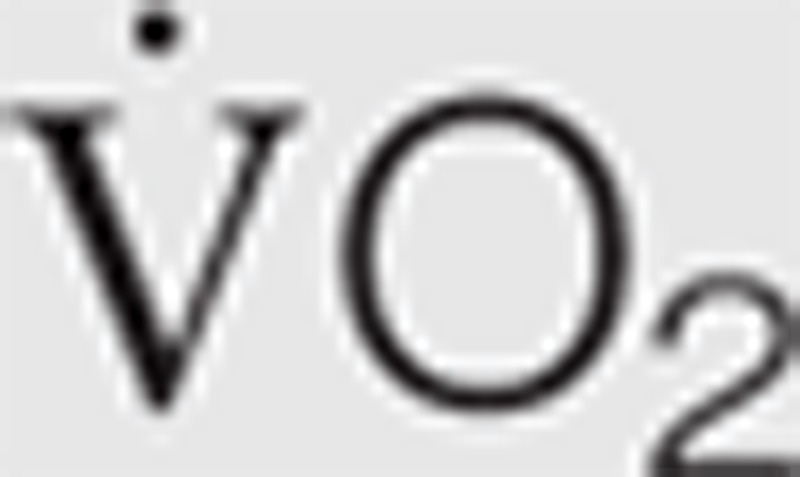
 peak (18.2 ± 4.1 vs 20.9 ± 6.6 mL/kg/min, *P* = .02), maximal 6-Minute Walking Distance (6MWD) (421 ± 90 vs 462 ± 74 m, *P* = .03), anaerobic threshold (12.4 ± 2.5 vs 13.4 ± 2.6 mL/kg/min, *P* = .005), and QOL. In summary, patients receiving home-based cardiac rehabilitation experienced a 14.2% increase in 
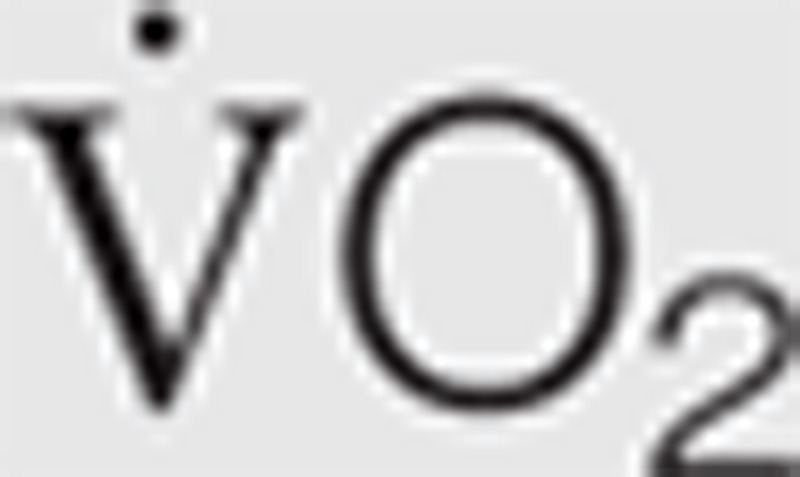
 peak, a 37% increase in QOL score, and an improvement of 41 m on the 6MWD test. The 90-day readmission rate for patients reduced to 5% from 14% after receiving cardiac rehabilitation.

**Conclusion:**

Home-based cardiac rehabilitation offered the most improved results in functional capacity, QOL, and a reduced the rate of readmission within 90 days.

## 1 Introduction

Heart failure (HF) is a common yet complicated disease resulting from multiple etiologies, including coronary artery disease, hypertension, and certain metabolic disorders, leading to over 10,000 deaths each year in Taiwan.^[1]^ HF is not curable and requires long-term evaluation and medical care. The incidence and prevalence rates of HF are increasing annually due to an aging populations, along with an increase in the prevalence rate of chronic systemic disorders, including hypertension, type 2 diabetes mellitus, and hyperlipidemia.^[2,3]^ The average age of patients with HF is also decreasing. The therapeutic goal of HF is to avoid any aggravating symptoms, improve health-related quality of life (QOL), and decrease the costs of health care.^[1,4]^

Although several reports have suggested that the mortality rate of patients with HF is improving, the overall mortality rate still remains high. It has been estimated that more than 23% of rehospitalizations for HF^[5]^ occur within 60 to 90 days, while less than 50% of patients with HF will survive for more than 5 years.^[5–7]^ According to the registry data of the American Heart Association (AHA) for Projections, the prevalence of HF will increase by^[7]^ 46% between 2012 and 2030, where patients with HF who are younger than 65 years old will have a 6 to 9 times greater risk of experiencing sudden cardiac death when compared with that of the general population.^[7]^ The huge medical expense of HF leads to a heavy economic burden on both the patient's family and the health care system. Previous studies have shown that some symptoms of HF, including fatigue and dyspnea on exertion, make the daily activities of patients with HF intolerable.^[3]^ Additionally, the aggravated symptoms of HF may cause depression, anxiety, and a compromised QOL for the patient.^[8–10]^

Several studies have shown that cardiopulmonary rehabilitation programs are both safe and effective for improving functional capacity and QOL, as well as for reducing the readmission rates and all-cause mortality of patients with HF.^[1,11,12]^ The results of many clinical trials have established the benefits of hospital-based cardiac rehabilitation for patients with HF.^[13–15]^ Home-based cardiac rehabilitation may be more accessible and acceptable when compared with hospital-based cardiac rehabilitation. However, home-based rehabilitation programs have not been widely studied and their training effects remain unclear.^[1,13,16]^ Thus, the purpose of this study was to evaluate the effects of home-based cardiac rehabilitation on the improvement in functional capacity, enhancement in QOL, and the reduction in the rate of readmission for patients with HF.

## 2 Methods

### 2.1 Study design

This study was a prospective randomized study, where a total of 75 patients participated from June 2013 to March 2014 in Taichung Veterans General Hospital. We explained in detail the purpose and methods of the study to all staff, including cardiologists, physical therapists, and nurses. HF patients with a reduced ejection fraction (HFrEF) from the general ward, the intensive care unit, along with outpatients from the department of cardiology, all taken from a single medical center in central Taiwan were included in this study. The chosen patients were well informed of the content of the study and were required to sign a consent form before joining the study. Patients were eligible to withdraw from the study at any time. We randomly assigned patients into the control group and interventional group. Data were scrambled before being made available to researchers in order to ensure that individual identifying information at any level could not be obtained from the database. Clinical data of patients in the study were collected by both nurses and case managers within the cardiology department. The study then proceeded after obtaining permission from the hospital's Institutional Review Board (IRB).

### 2.2 Study subjects

HF patients in either the ward or outpatient department with a left ventricle ejection fraction (LVEF) of less than 50% were included in this study. We evaluated the functional stage of HF using the New York Heart Association Functional Classification (NYHA Fc) guidelines. Patients experiencing NYHA Fc IV, pregnancy, a high bedridden status, musculoskeletal system problems, and disabilities for which exercise is contraindicated, were excluded from this study. HF patients with a preserved ejection fraction (LVEF >50%) were excluded from this study due to a difficulty with performing a dedicated evaluation.

### 2.3 Measurements

All patients had to sign a consent form before joining this study. We recorded each patient's general data, including body height, body weight, and laboratory data during their admission and outpatient visits. We then evaluated each patients’ QOL using the Minnesota Living With HF Questionnaire (*MLHFQ*) during the study period. We also monitored several parameters including 
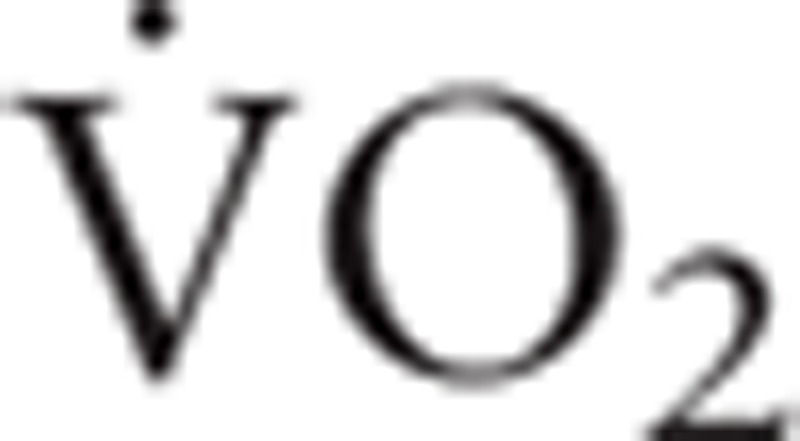
 peak, anaerobic threshold (AT) through use of the Cardiopulmonary Exercise Test (CPET) and the 6 Minutes Walking Test (6MWT) in order to evaluate the change exercise tolerance during the study period. According to the data obtained from both the CPET and 6MWT, we designed an individualized home-based cardiac rehabailitation program according to each patient's willingness. In addition, we monitored the parameters of their hemodynamics status, including stroke volume (SV), LVEF, and thoracic fluid index (TFI), by using a noninvasive cardiac output monitor (Aesculon [Osypka Medical, Berlin, Germany]) during the study period. We measured the patients’ general data and physiologic parameters in both groups at the beginning of the study and also 3 months later. Finally, we compared all collected data from both groups to evaluate both the change of exercise tolerance and QOL in the patients. Medications were not changed in any patient during the course of the study.

### 2.4 Exercise training protocol

In the interventional group, we collected general data and parameters using the same methods as we did for the control group. In addition, patients in the interventional group received outpatient cardiac rehabilitation for 1 week, before starting home-based cardiac rehabilitation. Home-based cardiac rehabilitation was conducted by requesting the interventional group to carry out aerobic exercise at least 3 times per week, for a duration of at least 30 minutes each time. Each patient was required to perform cardiac rehabilitation with an intensity measuring 60% to 80% of peak heart rate, based on the results of his or her initial CPET. The required exercise intensity was measured subjectively using a Borg score of 12 to 13.^[17]^ The types of exercises prescribed were based upon individual interests and abilities, and included walking (47.3%), jogging (5.4%), and stationary cycling (47.3%). The control group was instructed to maintain both their standard medical care and previous activity levels.

Regular home-based cardiac rehabilitation was to be performed for at least 3 months in the interventional group, and all data including CPET and 6MWD were collected after completion of the home-based cardiac rehabilitation. Medical education regarding HF was also provided by the nursing staff during admission and the case manager in the outpatient department both groups. We monitored patients through telephone interviews held every 2 weeks during the study period.

### 2.5 Statistical analysis

After collecting all data from patients in the study, it was then expressed as mean ± SD. Continuous variables were analyzed using 2-way analysis of variance (ANOVA), and a paired *t* test was used to compare group differences with baseline values. A *P* value <.05 was considered statistically significant. Calculations and statistical analyses were carried out using SPSS version 18.0 (SPSS Inc., Chicago, IL).

## 3 Results

Forty patients were randomly assigned to the control group, while 35 patients were randomly selected for the interventional group. In the control group, 3 patients died during the study period, 8 were lost during follow-up, while 11 had incomplete data at the end of the study. As a result, this total of 22 patients in the control group were excluded from the study. In the interventional group, 3 patients could not complete the cardiac rehabilitation course, 4 patients refused to receive the final test, and 9 were lost to follow-up. Therefore, a total of 16 patients in this group were excluded from the study. In the end, there were 18 HF cases in the control group and 19 HF cases in the interventional group. Within the interventional group, 6 of the 19 patients experienced ischemic cardiomyopathy, 2 patients had received coronary artery bypass grafting (CABG) surgery, while 9 of the 19 patients had received cardiac resynchronization therapy (CRT) before the start of the cardiac rehabilitation program. Within the control group, 3 of the 18 patients experienced ischemic cardiomyopathy, no patient had undergone CABG surgery, while 8 of 18 patients had received CRT. One patient in the control group had rheumatic heart disease with severe mitral stenosis and had received a mitral valve replacement before joining the study.

Table 1 summarizes the baseline characteristics of the patients. There were no statistically significant differences in age, NYHA Fc, etiology of HF, LVEF, 
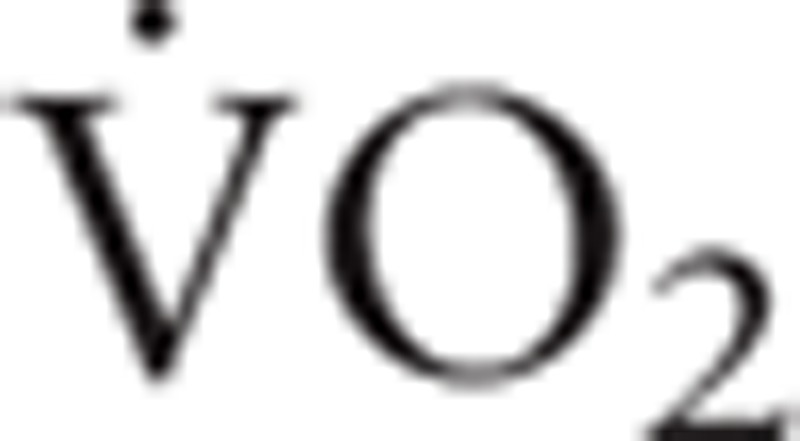
 peak, AT, or metabolic equivalent (MET) between the control and intervention patients.

**Table 1:**
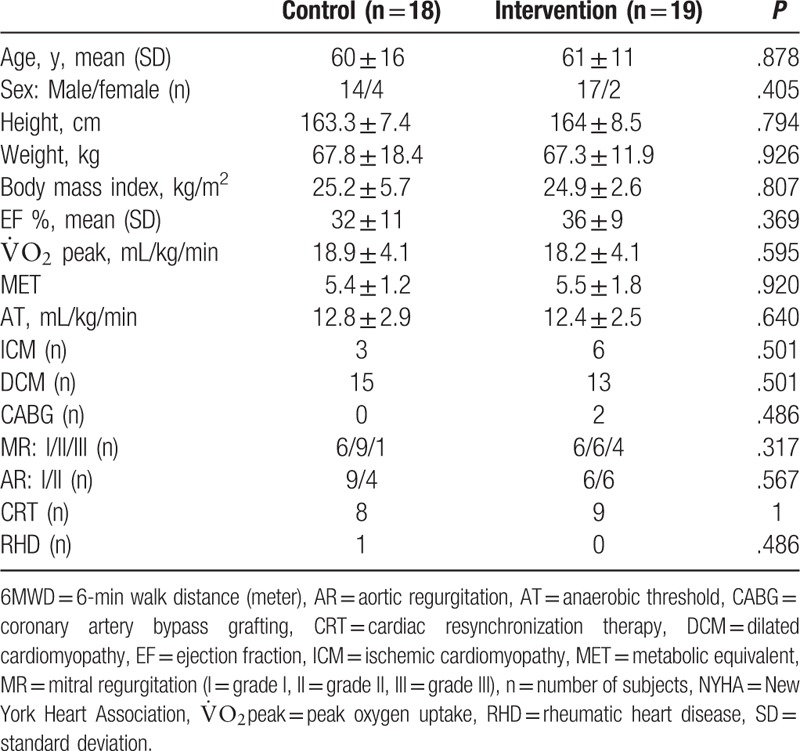
Baseline characteristics of enrolled patients.

In the interventional group, the patients who had participated in the home-based cardiac rehabilitation program showed a significant improvement of 
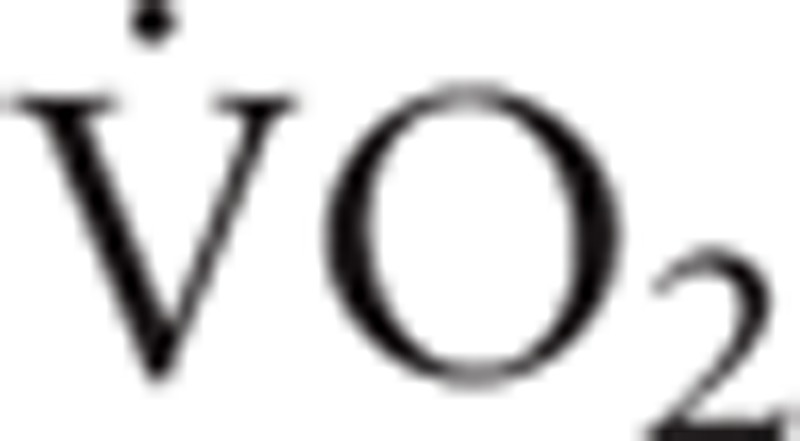
 peak by a margin of 14.2% (18.2 ± 4.1 vs 20.9 ± 6.6 mL/kg/min, *P* = .02), when compared with baseline. The *MLHFQ* score and 6MWD also increased significantly by the amount of 37% (32.1 ± 10.8 vs 20.2 ± 8.6, *P* < .01), and 41 m in the interventional group (421 ± 90 vs 462 ± 74 m, *P* = .03), respectively. The AT of the interventional group also improved remarkably (12.4 ± 2.5 vs 13.4 ± 2.6 mL/kg/min, *P* = .005). In the control group, there were visible declines in both 
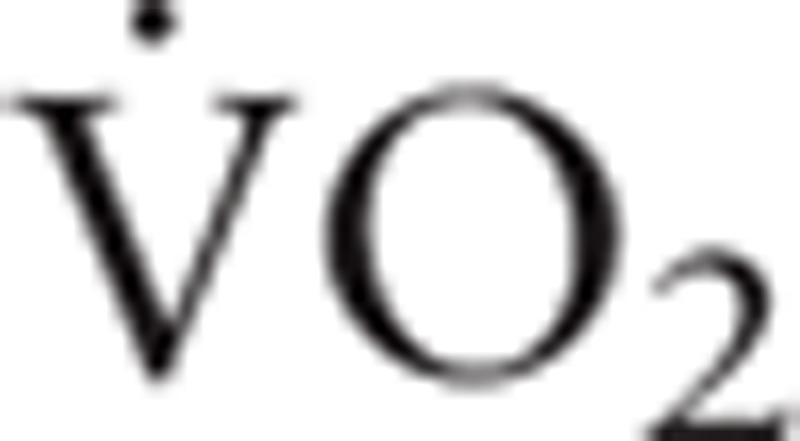
 peak and MET, but there were no notable changes in AT, 6MWD, and *MLHFQ* scores at the 3-month follow-up. Table 2 displays the changes in exercise tolerance and QOL in both groups.

**Table 2:**
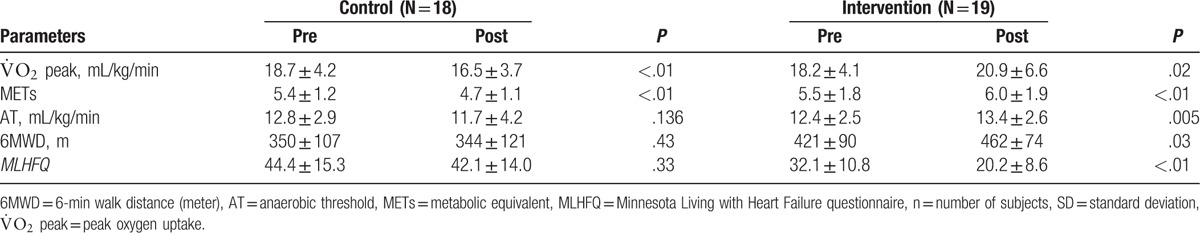
The changes of exercise tolerance and quality of life in both groups.

Data from the noninvasive cardiac output monitor, cardiac index (CI), SV, TFI, Left Ventricle Contractility Index (ICON), and systemic vascular resistance (*SVR)* were measured. The data showed a significant decline in the TFI of both groups after 3 months of training (26.5 ± 4.4 vs 22.5 ± 4.1 1/kΩ, *P* = .001 for the interventional group, 27.2 ± 6.8 vs 22.2 ± 3.8 1/kΩ, *P* < .01 for the control group). Other parameters showed no remarkable differences between the 2 groups. Table 3 summarizes the changes in the parameters of heart function in both groups.

**Table 3:**
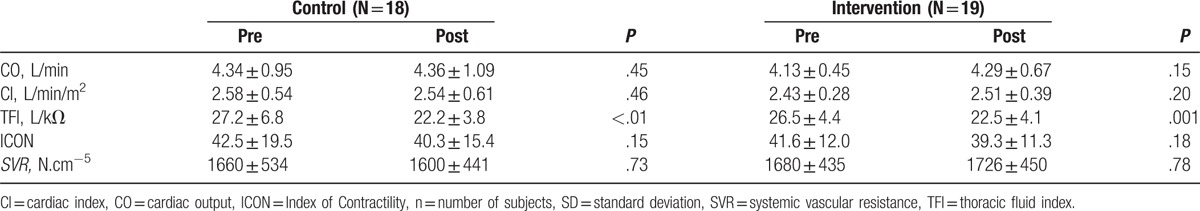
The change in parameters of heart function in both group.

According to data obtained from our hospital's database of patients’ medical records, the readmission rate for HF within 1 year was 34%, and 14% within 90 days during the period 2011 to 2012. At the 3-month follow-up period, the interventional group showed a significant reduction in the readmission rate within 90 days, decreasing from the average rate of 14% to 5%. The home-based cardiac rehabilitation program thus lowered the readmission rate for HF by nearly 10% for this 90-day follow-up period.

In conclusion, home-based cardiac rehabilitation programs can not only improve a patient's aerobic capacity, but they can also lower the readmission rate of patients with HF. Furthermore, no adverse events were reported during the home-based rehabilitation program.

## 4 Discussion

### 4.1 Major findings

Our study demonstrates that home-based cardiac rehabilitation results in a statistically significant improvement in both 
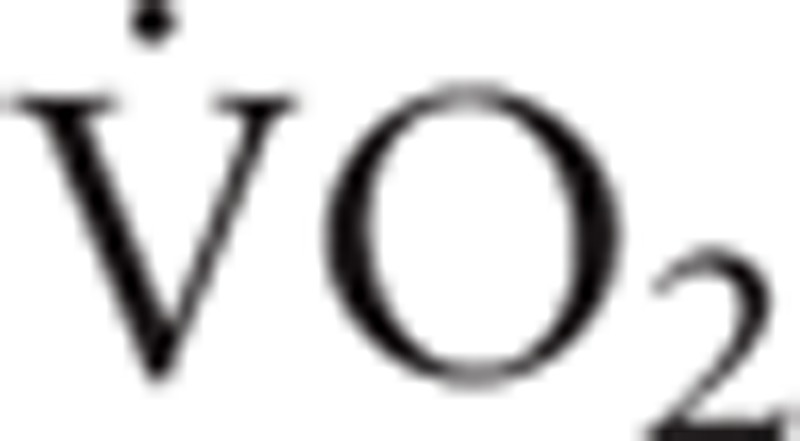
 peak and AT, which in turn was associated with improvements in functional capacity and QOL.

### Improvement in 
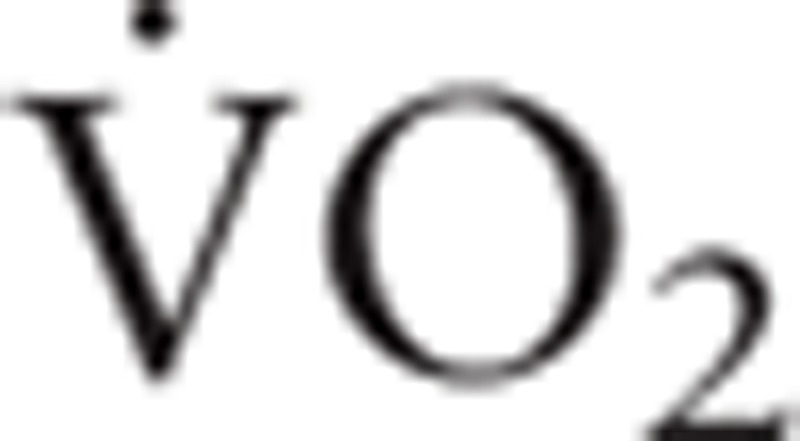
 peak and anaerobic threshold

4.2

In our study, home-based cardiac rehabilitation was associated with a remarkable improvement in 
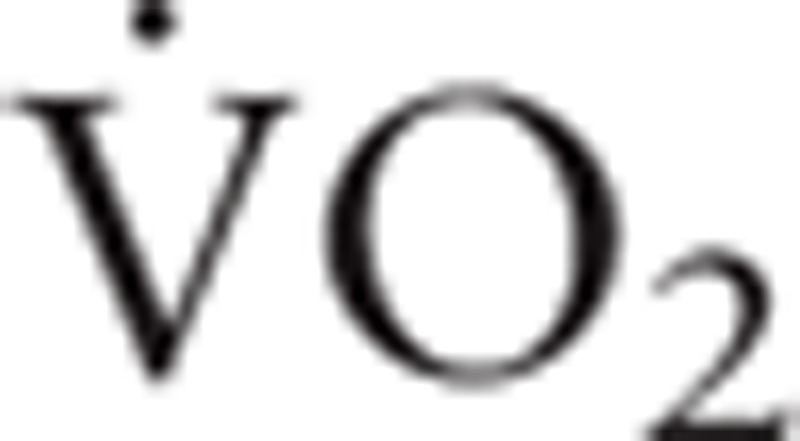
 peak, AT, and QOL. That same result could also be observed in outpatient-based cardiac rehabilitation.^[18,19]^ The improvement exercise tolerance patients with HFrEF can be well-explained by the improvement 
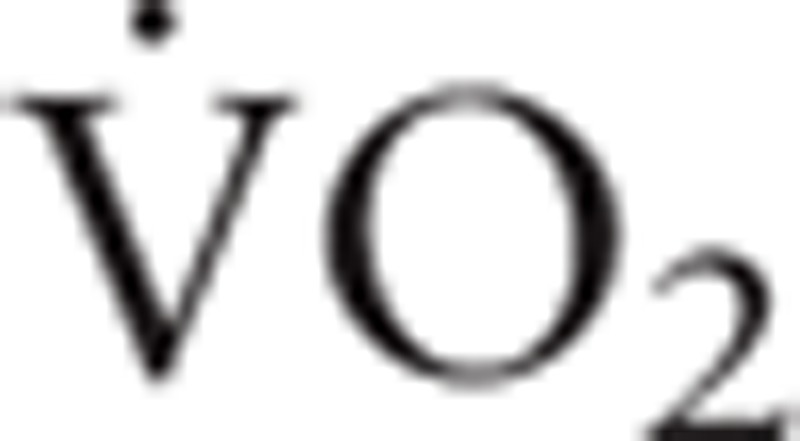
 peak and AT. Nevertheless, previous studies have shown that approximately 20% to 50% of patients with HF are unable to comply with hospital-based cardiac rehabilitation programs in the first 3 to 6 months.^[20]^ As a result, home-based cardiac rehabilitation programs are shown to be more convenient, and may be an acceptable alternative option for patients with chronic HF.^[21]^ Such home-based programs may therefore be a more practical strategy for motivating patients to continue exercise.^[22]^ Several published studies have shown that home-based exercise training could improve both 
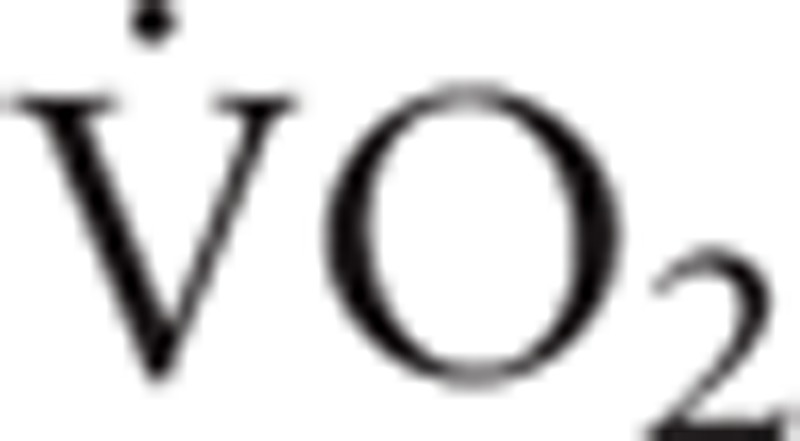
 peak and 6MWD.^[1]^ This is similar to our results, which showed an improved 
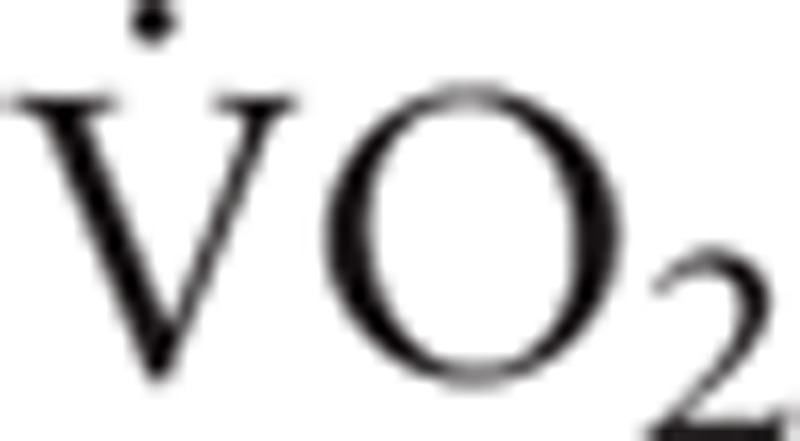
 peak of 2.7 mL/kg/min, an improved AT of 1.2 mL/kg/min, along with an improvement in 6MWD of 41 m.

### 4.3 Improvement in QOL

Several previous studies have shown that rehabilitation programs can lead to a statistically significant improvement in QOL for patients with HF.^[20,23,24]^ However, it remains controversial whether home-based cardiac rehabilitation benefits QOL or not. According to the results of our study, we observed that patients in the interventional group showed a significantly improved QOL after 3 months’ follow-up, compared with the control group. The improvement in QOL is also related to the improvement in exercise tolerance. In addition to the benefits that cardiac rehabilitation provides, a further advantage it has is the easy integration of a home-based cardiac rehabilitation into a patient's life. A home-based rehabilitation program has a lower impact on a patient's daily life. In contrast, there was a decrease in 
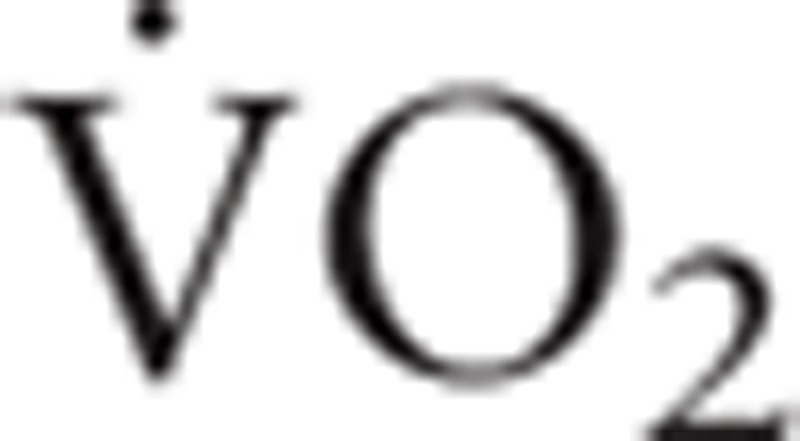
 peak and AT in the control group, which did not show any improvement in QOL. These data may explain, at least in part, why patients enrolled in the home-based cardiac rehabilitation program displayed a better QOL.

### 4.4 Improvement in 6-minute walking distance (6MWD)

The 6MWD has been proposed as an easy, well-tolerated, and alternative method for evaluating functional capacity.^[25]^ Previous studies have demonstrated that higher rates of death and hospitalization were found in HF patients with a 6MWD of less than 300 m.^[26]^ We showed that a 6MWD greater than 300 m may indicate a better prognosis. The meta-analysis offered strong evidence that the 6MWD was responsive to change in clinical status following cardiac rehabilitation, with an estimated mean difference in distance of 60.43 m.^[27]^ In our study, 6MWD results improved from 420 to 461 m after patients received home-based cardiac rehabilitation for 3 months. This increased distance of 41 m on the 6MWD test was associated with an increase in both 
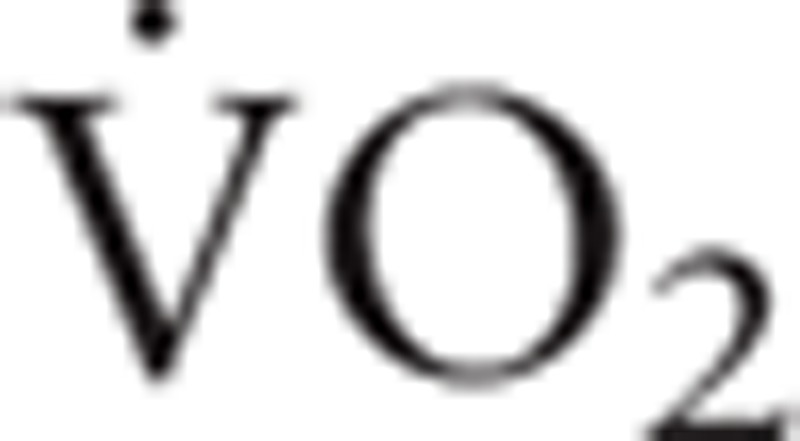
 peak and AT, after the home-based rehabilitation program.

### 4.5 Changes in heart function after rehabilitation

Previous studies have shown that exercise training offers no benefits toward heart function, including cardiac output, SV, and LVEF.^[28,29]^ Only 1 published study showed there was significant improvement in LVEF in both hospital-based and home-based exercise programs.^[30]^ However, the noninvasive cardiac output measurement data showed no significant change in our study. The results of this study were thus similar to the findings of previous research. Short-term, home-based cardiac rehabilitation no significant benefits to cardiac physiologic function. However, more long-term research is still required in order to evaluate the effects of such exercise programs on cardiac physiologic function. In our study, both groups experienced a lower TFI at 3 months, compared with that at the beginning of the study. Traditional treatment for HF, including medical therapy, diet education, and lifestyle modification, still provided benefits toward the control of fluid status in patients with HF. Although short-term rehabilitation had no effect on heart function, the improvement in both 
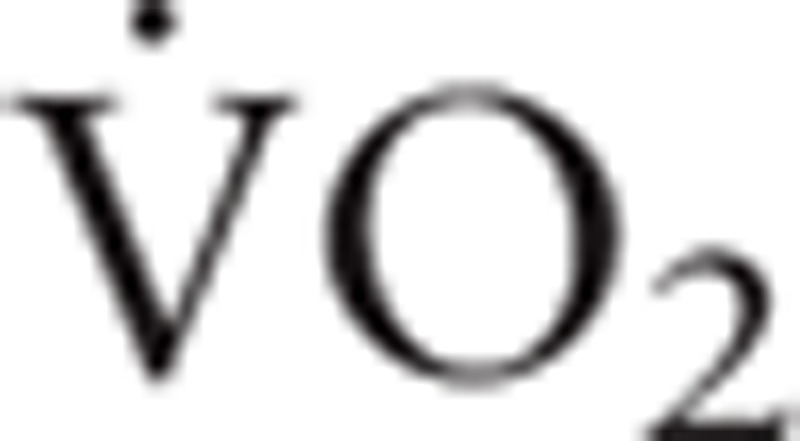
 peak and AT may play an important role in functional capacity and QOL.

### 4.6 Limitations

A limited number of subjects, high rate of loss follow-up, and a predominantly male subject pool are the limitations within this study. In addition, the study period may have been too short to see the full benefits of cardiac rehabilitation on the improvement of heart function. Although our study shows the benefits home-based cardiac rehabilitation has on exercise tolerance and QOL, further long-term study is still needed in order to show the effects it has on heart functions.

## 5 Conclusion

Home-based cardiac rehabilitation increased 
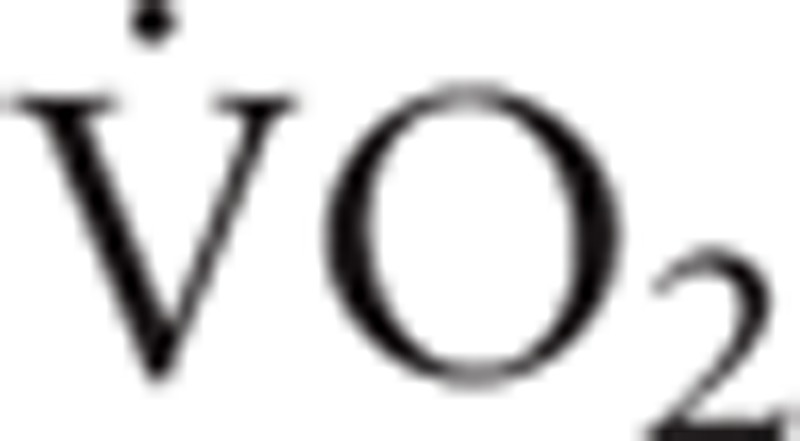
 peak by 14.2%, QOL by 37%, and 6MWD by 41 m, and reduced the rate of hospital readmission within the initial 90-day follow-up.
